# Calendar

**DOI:** 10.1155/S0962935195000627

**Published:** 1995-09


Mediators of Inflammation 4, 368–373 (1995)

**Tumour necrosis factor-α (TNF-α) was measured by enzyme-linked immunosorbent assay and eosinophil cationic protein (ECP) by radioimmunoassay to evaluate TNF-α in nasal allergy. There was no significant difference either between the mean concentrations of TNF-α in nasal secretions from the patients with perennial nasal allergy and those of normal subjects, or between the TNF-α and ECP concentrations. However, reverse transcription polymerase chain reaction showed a specific increase of TNF-α mRNA and IFN-γ mRNA in allergic nasal mucosa after allergen challenge *in vitro*. These findings suggest a possibility that T cell-derived IFN-γ up-regulates macrophages to elaborate TNF-α, which may play a role in amplifying allergic inflammation in the nose through the cytokine network.**

**Key words:** ECP, IFN-γ, mRNA, NIH image, rhinitis, RT-PCR, TNF-α.

### Tumour necrosis factor-α in nasal allergy

K. Hisamatsu,[1,CA] T. Ganbo,[1] T. Nakazawa,[1] T. Nakajima,[1] J. Ko,[1] R. Goto,[1] Y. Murakami[1] and K. Mitsui[2]

[1]Department of Otorhinolaryngology and [2]Second Department of Biochemistry, Yamanashi Medical University, 1110 Shimokato, Tamaho-cho, Nakakoma-gun, Yamanashi-ken 409-38, Japan

[CA]Corresponding Author

### Introduction

Tumour necrosis factor-α (TNF-α) was identified as an anti-tumour cytokine derived from macrophages (Mϕ).[1] So far, the sources of TNF-α include Mϕ, activated T cells,[2] and mast cells.[3,4] TNF-α has been recognized as having various biological activities relevant to inflammation, such as expressing adhesion molecules on endothelium,[5] being chemoattractant,[6] and enhancing microvascular permeability.[7] Recently, an increase of TNF-α in bronchoalveoral lavage fluids has been reported and recognized as an important mediator in bronchial asthma.[8] However, there have not been sufficient studies to clarify the role of TNF-α in nasal allergy. It has been reported that T cells elaborate interferon-γ (IFN-γ) to activate Mϕ.[9] Furthermore, recently, we elucidated the accumulation of activated helper T cells in the nasal mucosa of allergy patients by measuring soluble interleukin-2 receptor (sIL-2R).[10] These reports further suggest that T cells may induce TNF-α elaboration by Mϕ and mast cells in allergic nasal mucosa through the cytokine network following allergen exposure.

To investigate TNF-α in nasal allergy in the present study, we measured TNF-α in the nasal secretions of allergic patients. We also investigated TNF-α mRNA and IFN-γ mRNA in the nasal mucosa using reverse transcription polymerase chain reaction (RT-PCR) after allergen challenge *in vitro*. Furthermore, to investigate the relationship between TNF-α and eosinophils, we measured eosinophil cationic protein (ECP) in nasal secretions.

### Materials and Methods

*Subjects:* We studied 27 patients (15 males and 12 females, ranging in age from 21 to 32 years) and eight normal volunteers (four males and four females, ranging from 23 to 31 years). Patients were diagnosed with perennial nasal allergy according to the following criteria: (1) perennially persistent and recurrent nasal symptoms consisting of sneezing attacks, watery nasal discharge and nasal obstruction; (2) positive eosinophilia in nasal smear test; and (3) house dust-positive intradermal reaction and/or IgE antibody against house dust mite, *Dermatophagoides farine* in the serum. All patients and normal subjects gave informed consent. None of the patients or controls had other inflammatory diseases and none received any medication prior to nasal lavage collection. For mRNA analysis, the nasal mucosa obtained at surgery from four patients with house dust mite allergy was used.

*Assessment of clinical severity of nasal allergy:* Nasal symptoms scored in a symptom diary were assessed and the clinical severity was determined according to Okuda's criterion.[11] Frequency of sneezing attacks, nose blowing and patient-assessed grade of nasal obstruction were scored, and symptom grades were determined as mild, mod-

© 1995 Rapid Science Publishers erate or severe. When one or more of these symptoms were severe, overall clinical severity was considered severe. When the symptoms were moderate or mild, overall severity was considered moderate. When all of three symptoms were mild, overall severity was considered mild.

*Nasal smear test*: Nasal smears were obtained prior to nasal lavage, and cytologically investigated using Hansel's staining procedure[12] and nasal cytograms were graded according to a semi-quantitative scale as reported previously.[13]

*Preparation for measurement of TNF-α, and ECP*: Nasal lavage fluids were obtained by washing with 20 ml of 0.9% saline solution containing 1 mM LiCl, pre-warmed to 37°C, using a 30 ml plastic syringe with minimal stimulation of the nasal mucosa. The total volume of recovered lavage fluid was measured and the nasal lavage fluid was centrifuged twice at 1350 × **g** for 10 min at 4°C. Sputolysin® (Behring Diagnostics La Jolla, CA, USA) was mixed with nasal lavage fluid at a volume ratio of 0.4/9.0, and immediately shaken for 1 min, and then 5.5% aprotinin (Sigma, MO, USA) was added at a volume ratio of 0.1/9.4, followed by immediate mixing for 1 min at room temperature. The fluid was allowed to stand for 30 minutes at room temperature, then centrifuged twice at 1350 × **g** for 10 min at 4°C and stored at −80°C until TNF-α and ECP were assayed.

The lithium (Li) concentration in the nasal lavage was measured by atomic emission spectrophotometry to calculate TNF-α and ECP concentrations in the nasal secretions. Li was used as an exogenous marker of nasal secretion allowing calculations to be made from small amounts of nasal secretion.[14] The TNF-α concentration (TNF-$\alpha_x$) was calculated by the following equation:

$$\text{TNF-}\alpha_x = \text{TNF-}\alpha_n \times \text{Li}_o/(\text{Li}_o - \text{Li}_n)$$

where TNF-$\alpha_n$ denotes the TNF-α concentration of the sample; $\text{Li}_o$, the Li concentration of the 0.9% NaCl for lavage; and $\text{Li}_n$, the Li concentration of the sample. The ECP concentrations were calculated by the same equation using ECP instead of TNF-α.

*Measurement of TNF-α by enzyme-linked immunosorbent assay*: Enzyme-linked immunosorbent assay (Quantikine™, R&D Systems, Inc. MN, USA) was used to detect TNF-α in nasal lavage fluids. The assay was performed in duplicate. Briefly, 200 µl of standard or sample was added to 50 µl of the diluent and incubated at 37°C for 2 h in a flat-bottomed 96-well microplate. Then 200 µl of TNF-α conjugate was added after washing three times, then incubated for 1 h at room temperature. Then 200 µl of substrate solution was added after washing three times, and incubated for 2 min at room temperature, followed by adding 50 µl of stop solution. The absorbance was measured at 450 nm using an automatic spectrophotometer (Titerteck Multiscan® PLUS MKII, Flow Laboratories Inc., CA, USA). The absorbance of samples was measured by the spectrophotometer after calibrating the equipment to 0 using the substrate blank. TNF-α concentration in the nasal secretion was calculated according to the above equation. The minimal detectable dose using a standard curve was 4.4 pg/ml.

*Measurement of ECP:* The nasal lavage fluids from 18 allergic patients and from eight normal subjects were radioimmunoassayed (Pharmacia ECP RIA kit®, Pharmacia Diagnostics AB, Uppsala, Sweden) to determine ECP concentrations as described elsewhere.[15] The minimal detectable ECP was 2 pg/ml.

*Reverse transcription polymerase chain reaction:*

Preparation of the nasal mucosa for RT-PCR. Mucosal specimens were obtained from the inferior turbinates of allergic patients at times of surgery. The specimens were cut into 4 mm × 4 mm pieces, and incubated with or without extract of *Dermatophagoides farine* at a concentration of 1 µg/ml for 15 min in RPMI 1640, and then incubated at 37°C for 3 h.

Extraction of RNA. RNA was extracted from the mucosal specimens using RNeasy™ kit (QIAGEN, CA, USA). After treatment with DNase (Promega, WI, USA) at a ratio of 1 unit/µg RNA, to clean up the RNA, the RNA product was treated with the RNeasy™ kit (QIAGEN, CA, USA).

Preparation of cDNA. The RNA was incubated at 65°C for 10 min. One µg of the RNA, 2 µl of 10 × PCR Buffer II, 2 µl of 25 mM $MgCl_2$ (Perkin–Elmer, NJ, USA), 4 µl of 2.5 mM d-NTPs (dATP, dCTP, dGTP and dTTP; Pharmacia, NJ, USA), 1 µl of 20 U/µl RNasin (Wako, Osaka, Japan), 1 µl of 100 pM random primer, 1 µl of 200 unit/µl of Mo-MLVReverse Transcriptase (Gibco BRL, NY, USA) were mixed and adjusted to 20 µl in total volume by adding DEPC–water. The reaction mixture was allowed to stand at room temperature for 10 min, then incubated at 42°C for 60 min, and at 95°C for 5 min in a water bath. The obtained reverse transcription solution (cDNA) was stored at −20°C.

Table 1. Sequences of primers used in RT-PCR

| Target genes | 5′ primers | 3′ primers | Size of PCR product, bp |
|---|---|---|---|
| β-actin | 5′-ATGGATGATGATATCGCC-3′ | 5′-ATGAGGTAGTCAGTCAGGT-3′ | 574 |
| TNF-α | 5′-CTGCTGCACTTTGGAGTGAT-3′ | 5′-CCTTGGTCTGGTAGGAGACG-3′ | 356 |
| INF-γ | 5′-AATGCAGGTCATTCAGATG-3′ | 5′-CTGGGATGCTCTTCGACCTC-3′ | 386 |

PCR. The mixture consisted of 2 μl of cDNA, 2.5 μl of 10 × PCR Buffer II, 3 μl of 25 mM $MgCl_2$, 2 μl of 2.5 mM dNTPs, 12.75 μl of DEPC–water, 1.25 μl of 20 μM 5′-primer and 1.25 μl of 20 μM 3′-primer (Table 1), 0.25 μl of AmpliTaq®DNA polymerase (Perkin–Elmer, NJ, USA) were incubated in the Gene Amp®PCR System 2400 (Perkin–Elmer, NJ, USA) at 94°C for 30 s, at 55°C for 30 s, at 72°C for 1 min with 32 cycles of PCR. Finally, the PCR mixture was incubated at 72°C for 10 min. The PCR product was stained with ethidium bromide, and electrophoresis was performed on 1.2% agarose and the gel photographed. The optical density of each band on the gel was analysed using an Epson TG-6000, a Macintosh IIci computer and a public domain NIH Image 1.52 program*. There was a linear relationship between the optical density (OD) and PCR cycles up to 35 cycles. The ratio of the OD of each mRNA/the OD of β-actin mRNA was determined to assess changes in mRNA expressions.

*Statistical analysis:* The Kruskal–Wallis test was used to evaluate the relationship between TNF-α values and clinical severity. To evaluate the differences between mean values of the two groups, Student's *t*-test was used. Significance was indicated at $p < 0.05$.

### Results

*TNF-α concentration in nasal secretions:* TNF-α was detected in nasal secretions from allergic patients ($n = 27$) and normal subjects ($n = 8$). The mean (± S.E.) values of TNF-α concentration in nasal secretions from allergic patients and normal subjects were 489.5 ± 118.6 pg/ml and 269.8 ± 62.4 pg/ml, respectively. There was no significant difference between the mean values of these two groups, although the mean concentration of TNF-α of patients with mild nasal allergy showed a significantly higher level than that of normal subjects ($p < 0.05$). There was no correlation between TNF-α concentration and clinical severity (Fig. 1).

FIG. 1. Correlation between TNF-α in nasal secretions and clinical severity. There was no correlation between TNF-α in nasal secretions and clinical severity in perennial nasal allergy patients, although the mean concentration in mild nasal allergic patients was significantly higher than that in normal subjects ($p < 0.05$).

*ECP in nasal secretions:* The mean (± S.E.) value of ECP concentration in the nasal secretions of allergic patients was 270.1 ± 68.5 μg/l ($n = 19$), and it was significantly higher than that of normal subjects (less than the minimal detection level of the assay, $n = 8$, $p < 0.01$). However, there was no correlation between ECP and TNF-α concentrations in the same nasal secretions ($r = 0.089$, Fig. 2).

FIG. 2. Comparison between TNF-α and ECP concentrations in nasal secretions. There was no correlation between TNF-α and ECP concentrations in nasal secretions.

*The program is written by Wayne Rasband at the US National Institutes of Health and is available on the Internet by anonymous ftp from zippy.nimh.nih.gov or on floppy disk, from NTIS, 5285 Port Royal Rd, Springfield, VA 22161, USA; part number PB93-504868.

Table 2. The ratios of the OD of mRNAs/the OD of β-actin mRNA in the nasal mucosa. The ratios of the OD of mRNAs of TNF-α and IFN-γ/that of β-actin showed significant differences from the controls 3 h after allergen challenge, although the base line values of neither mRNA showed any significant differences between the groups with and without allergen challenge ($n=4$). There were decreases in the OD ratios of IFN-γ and controls after allergen challenge because of increased OD of β-actin mRNA.

| | | Baseline mean ± S.E. | | After 3 h mean ± S.E. | |
|---|---|---|---|---|---|
| TNF-α mRNA | control | 0.183 ± 0.016 | NS | 0.129 ± 0.032 | $p < 0.01$ |
| | allergen challenge | 0.214 ± 0.001 | | 0.343 ± 0.045 | |
| IFN-γ mRNA | control | 0.206 ± 0.063 | NS | 0.116 ± 0.024 | $p < 0.05$ |
| | allergen challenge | 0.292 ± 0.097 | | 0.262 ± 0.048 | |

FIG. 3. Effect of allergen challenge on TNF-α mRNA and IFN-γ mRNA in the nasal mucosa. The TNF-α mRNA and IFN-γ mRNA were increased in the nasal mucosa by specific allergen challenge *in vitro*. The figure shows expression of TNF-α mRNA and IFN-γ mRNA in allergic nasal mucosa after 3 h incubation following challenge with *Dermatophagoides farine* extract. Lane M, DNA molecular weight marker; Lane 1, β-actin (without allergen challenge); Lane 2, β-actin (with allergen challenge); Lane 3, TNF-α (control); Lane 4, TNF-α (with allergen challenge); Lane 5, IFN-γ (control); Lane 6, IFN-γ (with allergen challenge). Ratios of the OD of TNF-α mRNA/the OD of β-actin mRNA and the OD of IFN-γ mRNA/the OD of β-actin mRNA were increased.

*Effect of allergen challenge on TNF-α mRNA and IFN-γ mRNA in the nasal mucosa:* RT-PCR revealed mRNAs of both TNF-α and IFN-γ in the nasal mucosa. The levels of TNF-α mRNA and IFN-γ mRNA 3 h after allergen challenge *in vitro* were significantly higher than those without allergen challenge, although there was no significant difference between the base line levels of either mRNA with allergen challenge and those of the controls ($n = 4$, Table 2, Fig. 3).

### Discussion

Specific reaction of the allergen with IgE antibodies bound to mast cells causes immediate and late nasal responses. The mast cells release chemical mediators and also elaborate cytokines such as IL-3,[16] IL-4, IL-5, IL-6, GM-CSF[17] and TNF-α.[18] These cytokines may take part in the network and modulate allergic inflammation of the nasal mucosa.

Eosinophil accumulation in the nasal mucosa is a hallmark of nasal allergy, and nonspecific hyperresponsiveness of the nasal mucosa is an important clinical feature of nasal allergy. Eosinophils predominantly release lipid mediators such as leukotriene $C_4$ ($LTC_4$)[19] and platelet activating factor (PAF)[20] as well as cytotoxic granule proteins such as major basic protein, ECP, eosinophil peroxidase and eosinophil derived neurotoxin.[21] Recent studies have suggested that eosinophils play an important role in the mechanism of mucosal nonspecific hyperresponsiveness. Therefore, eosinophil recruitment in the nasal mucosa is crucial in the pathogenesis of nasal allergy.

In the present study, TNF-α was detected in nasal lavage fluid from patients with perennial nasal allergy. Bachert *et al.*[22] reported that TNF-α increased in nasal lavage during immediate nasal response. Some previous studies have reported that this cytokine was detected or not detected in nasal lavages from nasal allergy patients.[23,24] Inconsistent results are presumably due to rapid metabolism of this cytokine in the nasal lavage fluids and/or the small amount present, so that the level was undetectable in some cases by ELISA. It is also surmised that TNF-α might be converted rapidly during transfer into the nasal secretions from the nasal mucosa following the allergic response. We used a protease inhibitor, aprotinin, in the nasal lavage fluid and this possibly resulted in a successful detection of TNF-α in the nasal lavage in the present study, although the level of TNF-α in nasal secretions did not show any relation either to clinical severity, or to the ECP level in nasal secretions. On the other hand, the ECP level in nasal secretions showed a positive relationship to clinical severity in our previous study.[15] Recently, human mast cells were found to contain preformed TNF-α which is capable of release by immunological activation.[3,4,23] These data suggest that TNF-α may be released and play a role in immediate nasal response.

TNF-α was reported to be derived from activated Mϕ. To date, it has been reported that not only Mϕ but also mast cells and T cells generate TNF-α, which has various biological activities such as promotion of: (1) oxygen radical production of eosinophils and neutrophils;[7] (2) mast cell and eosinophil cytotoxicity;[25,26] (3) microvascular permeability;[7] (4) leukotriene $B_4$ ($LTB_4$) and PAF generation from eosinophils and neutrophils;[27,28] and (5) eosinophil and neutrophil adhesion to the endothelium and also airway epithelium by expression of adhesion molecules.[5,29] Furthermore, it is a chemoattractant for neutrophils and monocytes.[6] These biological activities lead to a hypothesis that TNF-α may amplify allergic inflammation resulting in mucosal hyperresponsiveness. Reports that TNF-α induces airway hyperresponsiveness in experimental animals[30,31] support this hypothesis.

It has been reported that TNF-α induces expression of adhesion molecules such as ICAM-1, VCAM-1 and ELAM-1 on vascular endothelial cells,[29,32,33,34] which may result in the adhesion of inflammatory cells to the endothelium and transmigration into the lesion. It is well known that $LTB_4$ and PAF are potent chemoattractants for eosinophils[35,36] despite the fact that these lipid mediators do not specifically act on eosinophils like IL-5.[37] Therefore, TNF-α might directly or indirectly be involved in late nasal response with infiltration of eosinophils and/or other inflammatory cells in allergic nasal mucosa by promoting the generation of these lipid mediators of inflammatory cells. Thus, to clarify whether TNF-α is generated or not after allergen challenge, the TNF-α mRNA in the allergic nasal mucosa was examined using specific RT-PCR because of presumable small amount of TNF-α in the culture supernatant.

Specific RT-PCR revealed TNF-α mRNA in the allergic nasal mucosa before and after allergen challenge *in vitro* in the present study. TNF-α mRNA specifically increased in the allergic nasal mucosa after allergen challenge. These data suggest that allergen exposure positively induces TNF-α generation in allergic nasal mucosa resulting in inflammatory cell recruitment. This hypothesis is supported by positive hybridization signals for TNF-α mRNA and a significant increase in the number of TNF-α mRNA-positive cells in the nasal mucosa of allergic patients after nasal allergen challenge.[38]

The present study also revealed that TNF-α concentrations in nasal secretions did not correlate with either clinical severity or concentrations of ECP in the nasal secretions obtained from allergic patients. As previously described, TNF-α has various biological activities relevant to accumulation and activation of inflammatory cells involved in allergic reactions. Consequently, these negative correlations suggest that TNF-α may indirectly play a role in causing clinical symptoms, and other factors may collaborate with TNF-α in eosinophil activation. In other words, TNF-α may play a central role in the cytokine network in the nasal allergy by regulating inflammatory cells such as lymphocytes, macrophages, mast cells and eosinophils.

Our previous studies suggested activation of helper T cells in nasal allergy[10] by elevation of sIL-2R level in nasal secretions and also cluster formation of CD25+ cells in the allergic nasal mucosa. Activated T cells are capable of generating IFN-γ, which is a Mϕ activating factor that promotes TNF-α and IL-1 production of Mϕ.[39] IFN-γ also promotes TNF action by increasing TNF receptor expression on sensitive cells.[40] In the present study, IFN-γ mRNA and TNF-α mRNA were determined in the same allergic nasal mucosa following allergen exposure. This result suggests a possibility that T cell-derived IFN-γ may take part in up-regulation of TNF-α amplifying sequential allergic events in the allergic nasal mucosa collaborating with TNF-α derived from other inflammatory cells such as T cells and mast cells. Details of cell-to-cell interaction through the cytokine network relevant to TNF-α remain to be clarified in nasal allergy.

### References

1. Old LJ. Tumor necrosis factor (TNF). *Science* 1985; **230:** 630–632.
2. Pennica D, Nedwin GE, Hayflick JS, *et al.* Human tumor nerosis factor: precursor structure, expression and homology to lymphotoxin. *Nature* 1985; **312:** 724–729.
3. Walsh LJ, Trinchieri G, Waldorf HA, Whittaker D, Murphy GF. Human dermal mast cells contain and release tumor necrosis factor-α, which induces endothelial leukocyte adhesion molecule-1. *Proc Natl Acad Sci USA* 1991; **88:** 4220–4224.
4. Gordon JR, Galli SJ. Mast cells as a source of both preformed and immunologically inducible TNF-α/cachectin. *Nature* 1990; **346:** 274–276.
5. Lamas AM, Mulroney CM, Schleimer RP. Studies on the adhesive interaction between purified human eosinophils and cultured vascular endothelial cells. *J Immunol* 1988; **140:** 1500–1505.
6. Ming WJ, Bersani L, Mantovani A. Tumor necrosis factor is chemotactic for monocytes and polymorphonuclear leukocytes. *J Immunol* 1987; **138:** 1469–1474.
7. Slungaard BA, Vercellotti GM, Nelson RD, Jacob HS. Tumor necrosis factor α/cachectin stimulates eosinophil oxidant production and toxicity towards human endothelium. *J Exp Med* 1990; **171:** 2025–2041.
8. Taki F, Kondoh Y, Matsumoto K, Takagi K, Satake T. Tumor necrosis factor in sputa of patients with bronchial asthma on exacerbation. *Arerugi* 1991; **40:** 643–646.
9. Broide DH, Lotz M, Cuomo AJ, Coburn DA, Federman EC, Wasserman SI. Cytokines in symptomatic asthma airways. *Allergy Clin Immunol* 1992; **89:** 958–967.
10. Hisamatsu K, Ganbo T, Nakazawa T, Horiguchi S, Shimomura S, Murakami Y. Elevation of soluble interleukin-2 receptor in nasal allergy. *Mediators of Inflammation* 1994; **4:** 39–42.
11. Okuda M. Basic and clinical study on nasal allergy. *Otolaryngol (Tokyo)* 1974; **20:** 297–344.
12. Hansel FK. Cytological diagnosis in respiratory allergy and infection. *Ann Allergy* 1966; **24:** 564–579.
13. Krause HF. Nasal cytology in clinical allergy. In: Krause HF, ed. *Otolaryngic Allergy and Immunology.* Philadelphia, USA: WB Saunders Company, 1989; 112–122.
14. Linder A, Venge P, Deuschel H. Eosinophil cationic protein and myeloperoxidase in nasal secretions as marker of inflammation in allergic rhinitis. *Allergy* 1987; **42:** 583–590.

15. Hisamatsu K, Ganbo T, Goto R, Nakazawa T, Murakami Y. Eosinophil cationic protein in perennial allergic rhinitis. *Auris/Nasus/Larynx* 1995; **22:** 165–171.
16. Ihle JN, Keller J, Oraslan S, *et al.* Biologic properties of homogenous interleukin 3. I. Demonstration of WEHL-3 growth factor activity, mast cell growth factor activity, P cell stimulating factor activity, colony stimulating factor activity and histamine producing cell-stimulating activity. *J Immunol* 1983; **131:** 282–287.
17. Plaut M, Pierce JH, Watson CJ, Hanley-Hyde J, Nordan RP, Paul WE. Mast cell lines produce lymphokines in response to cross-linkage of FcεR1 or to calcium ionophores. *Nature* 1989; **339:** 64–67.
18. Bradding P, Roberts JA, Britten KM, *et al.* Interleukin-4, -5, and -6 and tumor necrosis factor-alpha in normal and asthmatic airways: evidence for the human mast cell as a source of these cytokines. *Am J Respir Cell Mol Biol* 1994; **10:** 471–480.
19. Kajita T, Yui Y, Mita H, Taniguchi N, Saito H, Mishima T, Shida T. Release of leukotriene $C_4$ from human eosinophils and its relation to the cell density. *Int Arch Allergy Appl Immunol* 1985; **78:** 406–410.
20. Lee T, Lenihan D, Malone B, Roddy LL, Wasserman SI. Increased biosynthesis of platelet-activating factor in activated human eosinophils. *J Biol Chem* 1984; **259:** 5526–5530.
21. Gleich GJ, Adolphson CR. The eosinophilic leukocyte: structure and function. *Advanced Immunology* 1986; **39:** 177–253.
22. Bachert C, Ganzer U. Die Rolle der proinflammatorischen Zytokine bei der Rekrutierung von Entzundungszellen an der Nase. *Laryngo-Rhino-Otologie* 1993; **72:** 585–589.
23. Bradding P, Mediwake R, Feather IH, Madden J, Church MK, Holgate ST, Howarth PH. TNFα is localized to nasal mucosal mast cells and is released in acute allergic rhinitis. *Clin Exp Allergy* 1995; **25:** 406–415.
24. Gosset P, Malaquin F, Delneste Y, Wallaert B, Capron A, Joseph M, Tonnel AB. Interleukin-6 and interleukin-1 alpha production is associated with antigen-induced late nasal response. *J Allergy Clin Immunol* 1993; **92:** 878–890.
25. Benyon RC, Bissonette EY, Befus AD. Tumor necrosis factor alpha-dependent cytotoxicity of human skin mast cells is enhanced by anti-IgE antibodies. *J Immunol* 1991; **147:** 2253–2258.
26. Silberstein DS, David JR. Tumor necrosis factor enhances eosinophil toxicity to *Schistosoma mansoni* larve. *Proc Natl Acad Sci USA* 1986; **83:** 1055–1059.
27. Roubin R, Elsas PP, Fiers W, Dessein AJ. Recombinant human tumor necrosis factor (rTNF) enhances leukotriene biosynthesis in neutrophils and eosinophils with the $Ca^{2+}$ ionophore A23187. *Clin Exp Immunol* 1987; **70:** 484–490.
28. Camussi G, Tetta C, Bussolino F, Baglioni G. Tumor necrosis factor stimulates human neutrophils to release leukotriene $B_4$ and platelet activating factor. *Eur J Biochem* 1989; **182:** 661–666.
29. Wegner CD, Gundel RH, Reilly P, Haynes N, Letts LG, Rothelin R. Intercellular adhesion molecule-1 (ICAM-1) in the pathogenesis of asthma. *Science* 1990; **247:** 456–459.
30. Wheeler AP, Jesmok G, Brigham KL. Tumor necrosis factor's effect on lung mechanics, gas exchange, and airway reactivity in sheep. *J Appl Physiol* 1990; **68:** 2542–2549.
31. Kips JC, Tavernier J, Pauwels A. Tumor necrosis factor (TNF) causes bronchial hyperresponsiveness in rats. *Am Rev Respir Dis* 1992; **145:** 332–336.
32. Pober JS, Gimbrone MA, Lapierre LA, Mendrick DL, Fiers W, Rothlein R, Springer TA. Overlapping patterns of activation of human endothelial cells by interleukin-1, tumor necrosis factor, and immune interferon. *J Immunol* 1986; **137:** 1893–1896.
33. Osborn L, Hession C, Tizard R, Vassallo C, Luhowskyi R, Chi-Rosso C, Lobb R. Direct expression and cloning of vascular cell adhesion molecule-1, a cytokine-induced endothelial protein that binds to lymphocytes. *Cell* 1989; **59:** 1203–1211.
34. Bevilacqua MP, Stengelin S, Ginbrone MA, Seed B. Endothelial leukocyte adhesion molecule-1: an inducible receptor for neutrophils related to complement regulatory proteins and lectins. *Science* 1989; **243:** 1160–1165.
35. Wardlaw AJ, Moqbel R, Cromwell O, Kay AB. Platelet activating factor: a potent chemotactic and chemokinetic factor for human eosinophils. *J Clin Invest* 1986; **78:** 1701–1706.
36. Sigal CE, Valone FH, Holtzman MJ, Goetzl EJ. Preferential human eosinophil chemotactic activity of the platelet activating factor (PAF) 1-O-hexadecyl-acetyl-*sn*-glyceryl-3-phosphocoline (AGEPC). *J Clin Immunol* 1987; **7:** 179–184.
37. Wang JM, Rambaldi A, Biondi ZG, Chen ZG, Sanderson CJ, Mantovani A. Recombinant interleukin 5 is a selective eosinophil chemoattractant. *Eur J Immunol* 1989; **19:** 701–705.
38. Ying S, Robinson DS, Varney V, *et al.* TNF alpha mRNA expression in allergic inflammation. *Clin Exp Allergy* 1991; **21:** 745–750.
39. Collart MA, Belin D, Vassalli JD, de Kossodo S, Vassalli P. γ-Interferon enhances macrophage transcription of the tumor necrosis factor/cachectin, interleukin 1, and urokinase genes, which are controlled by short-lived repressors. *J Exp Med* 1986; **164:** 2113–2118.
40. Aggarwal BB, Essalu TE, Hass PE. Characterization of receptor for human tumor necrosis factor and their regulation by γ-interferon. *Nature* 1985; **318:** 665–667.

**Received 24 May 1995;**
**accepted in revised form 13 July 1995**
